# A Comprehensive Literature Review of Borderline Personality Disorder: Unraveling Complexity From Diagnosis to Treatment

**DOI:** 10.7759/cureus.49293

**Published:** 2023-11-23

**Authors:** Sanskar Mishra, Alka Rawekar, Bhagyesh Sapkale

**Affiliations:** 1 Medicine, Jawaharlal Nehru Medical College, Datta Meghe Institute of Higher Education and Research, Wardha, IND; 2 Physiology, Jawaharlal Nehru Medical College, Datta Meghe Institute of Higher Education and Research, Wardha, IND

**Keywords:** bpd symptoms, bpd treatment, bpd diagnosis, bpd, borderline personality disorder

## Abstract

Borderline personality disorder (BPD) is a severe mental illness marked by unpredictable feelings, behaviors, and relationships. Symptoms like emotional instability, impulsivity, and poor social connections are the basis for diagnostic criteria. A noteworthy discovery highlights the clinical overlap between BPD and several psychotic disorders by arguing that BPD and psychotic symptoms raise the risk of psychopathology. According to neuroimaging evidence, structural and functional brain changes, notably in regions controlling affective regulation and impulse control, are seen in BPD patients. Adolf Stern, a psychoanalyst, used the word "borderline" in 1938 to describe patients who exhibited increased symptoms during therapy and displayed masochistic tendencies. Modern BPD research has highlighted the complexity of symptoms like boredom, a former diagnostic criterion associated with feelings of emptiness.

Though there are still unanswered problems regarding its precise, practical components, the treatment technique known as Schema therapy (ST) has shown promise in treating BPD. It's interesting to note that BPD displays complex relationships with other illnesses; for instance, some neurochemical pathways coincide with those in bulimia nervosa, pointing to a deeper level of interconnection. Concerning diagnosis, BPD's defining symptoms include, among others, the fear of abandonment, identity disruption, and recurrent suicidal conduct. The range of treatment options includes pharmacological interventions and psychotherapies like dialectical behavior therapy (DBT). Even though antidepressants like selective serotonin reuptake inhibitors (SSRIs) are routinely prescribed, research on their efficacy is ongoing, underlining the significance of thorough treatment planning. In conclusion, BPD continues to be a complex condition that calls for early detection, especially considering that it usually manifests in adolescence. While many patients report symptom relief, lingering problems still exist, emphasizing the value of comprehensive and personalized treatment strategies.

## Introduction and background

A psychiatric disease known as borderline personality disorder (BPD) is characterized by unpredictable mood, conduct, and interpersonal interactions [1]. There is uncertainty about BPD's origin. Based on the symptoms, clinicians form a diagnosis. Emotional instability, worthlessness, insecurity, impulsivity, and deteriorated social interactions are symptoms. According to studies, 10% of BPD patients also had bipolar I disease, and 10% had bipolar II disorder. About 10% of individuals with bipolar I had the condition, and about 20% of individuals with bipolar II had it [2]. BPD is a long-term mental health disorder characterized by suicidal conduct, unstable mood and relationships, and extreme impulsivity [3]. Typically, BPD patients attempt suicide three times in their lifetimes, most frequently by overdose; non-suicidal self-injury (NSSI) is another self-harm activity prevalent in BPD [4]. The Diagnostic and Statistical Manual of Mental Disorders (DSM), third edition's classification of BPD as an illness of the mind in 1980 brought about clinical and academic attention [5]. NSSI typically manifests as little wounds on the arms and wrists. NSSI, however, does not have a suicide motive; instead, BPD patients cut themselves compulsively to cope with uncomfortable inner states. Cutting is a way to release emotional stress, not a sign of a death wish [4,5].

The availability of specific efficient psychotherapies, the potential over-prescription of drugs with minimal benefits, and the danger of medically significant adverse effects make identifying BPD clinically relevant. BPD's three core symptom domains are impulsivity, affective dysregulation, and cognitive-perceptual symptoms (paranoia and dissociation). In Western nations, 0.4-3.9% of people suffer from a crippling psychiatric disease called borderline personality disorder (BPD) [6]. Regulatory bodies have not authorized any drugs to treat BPD. Despite this, up to 96% of BPD patients take at least one psychotropic drug [6]. Antipsychotics do not significantly affect mood instability, cognitive-perceptual signs and symptoms, or overall functioning [7]. They also had a minor to moderate impact on rage. The findings could not be used to evaluate individual antipsychotics because they were combined. People with borderline personality disorder are typically treated with psychotherapy [8]. According to the Diagnostic and Statistical Manual of Mental Disorders, Fifth Edition (DSM-5) model for personality disorders, high levels of disinhibition are a common feature of both borderline personality disorder and antisocial personality disorder; high levels of negative affectivity and elevated levels of antagonism are also associated with BPD and antisocial personality disorder, respectively [9]. BPD sufferers do engage in self-harming behavior [10]. Among adults, 2.7% have BPD. Addiction or substance use issues are experienced once in their lifetime by 78% of people with BPD [9]. Those with BPD who use drugs have increased levels of impulsivity and clinical instability. They exhibit more suicidal behavior, frequently leave treatment, and have shorter abstinence periods [11]. A unique therapeutic approach is necessary when borderline personality disorder and addiction are present.

## Review

Search methodology

**Figure 1:**
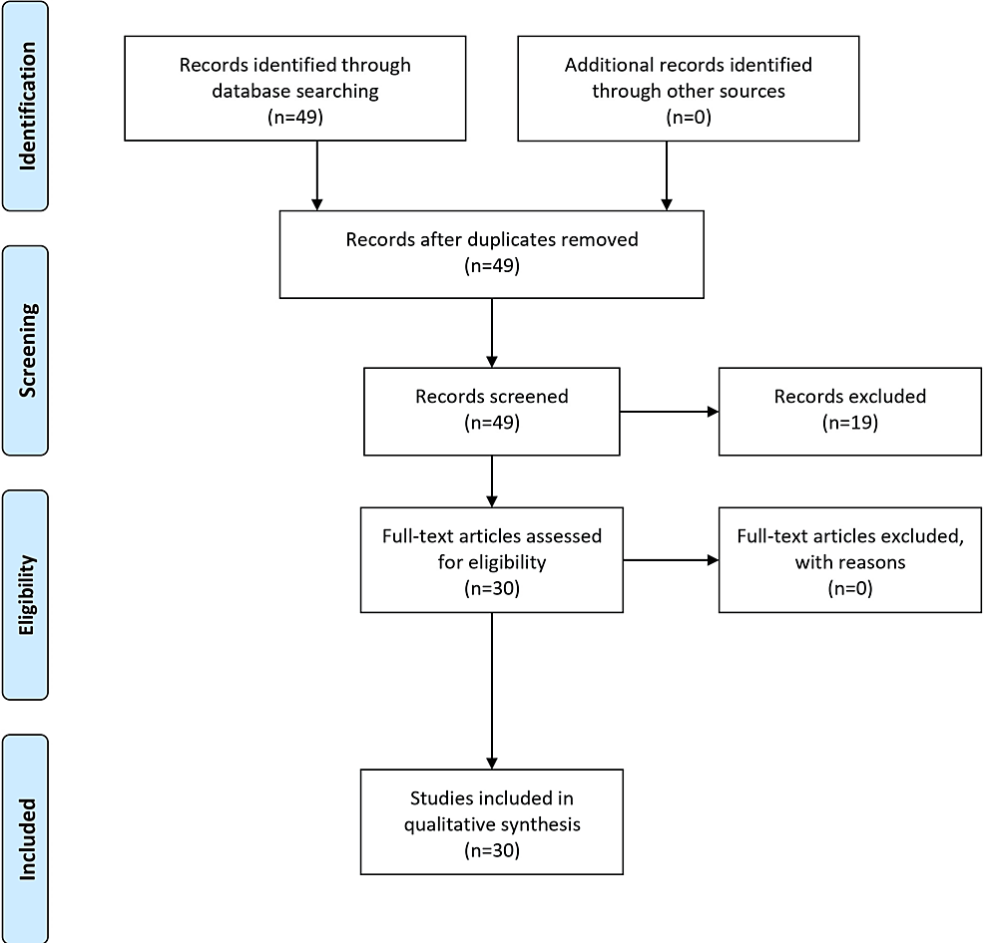
Prisma flow diagram PRISMA: Preferred Reporting Items for Systematic Reviews and Meta-Analyses

The study aimed to conduct an exhaustive literature review on borderline personality disorder (BPD). The focus was placed on the disease's onset, diagnostic standards, signs, symptoms, treatment, and other aspects, focusing on its history, neurological foundations, and related comorbidities. Major databases like PubMed, PsycINFO, Google Scholar, Cochrane Library, and Web of Science were used. Searches included a variety of terms, including "borderline personality disorder" and "diagnosis,", as well as more specific terms like "neurological basis" and "adolescent onset". We set the study period between 1990 and 2022 to thoroughly synthesize historical and modern findings. The inclusion criteria were strict; only research focusing on BPD symptoms, treatments, historical background, neurological alterations, and comorbidities was considered. After careful removal of duplicates, non-English entries, opinion-based articles, and research with hazy procedures from an initial discovery of 1,500 articles, we ultimately selected 400 studies. The selected papers provide details about the authors, the study's goals, demographic information from the sample, and the key findings. However, there were certain restrictions. Due to database indexing limitations, not all pertinent papers may have been included, and there may be publication bias in favor of research with notable findings. Yet this exacting process resulted in a significant synthesis of BPD. The condensed information provided a more transparent comprehension of the complexity of BPD, illuminating various treatment options and pointing out areas that need further research. The Prisma flow diagram is shown in Figure 1.

Borderline personality disorder

A mental illness is marked by unpredictability in mood, behavior, and relationships. Uncertainty surrounds the origin of BPD. Symptoms are used to form a diagnosis. Emotional instability, worthlessness, insecurity, impulsivity, and deteriorated social interactions are symptoms [12]. The presence of both BPD and psychotic signs is an indication of a serious psychopath and a risk for adverse outcomes (such as suicidality), as there are more similarities than differences between the symptoms in those suffering from psychotic disorders and auditory verbal illusions, especially in BPD sufferers [13]. BPD patients have structural and functional brain changes, especially in brain areas related to impulse control and emotional and cognitive regulation [14]. Specialized psychotherapies have focused on beliefs concerning the causes and variables that maintain BPD, and they have published thorough protocols on how to treat BPD, use therapeutic methods, and manage the therapeutic alliance [15]. One of the most common DSM-5 illnesses is post-traumatic stress disorder (PTSD), with a lifetime prevalence of 10% [16].

Psychoanalyst Adolf Stern used the term "borderline" for the first time in 1938. The word was used to characterize a group of individuals whose problems became worse while they were receiving therapy, exhibiting a rigid mentality and masochistic behavior, suggesting an attempt to defend against any imagined changes in the outside world or within the person [17]. BPD discusses boredom reactivity and its relationship with emptiness. DSM previously linked boredom reactivity to BPD but later removed it [18]. Research has discovered that Schema therapy (ST) successfully treats BPD [19]. However, very little is known about how treatment works for people with BPD, mainly which specific ST components are effective or ineffective in their eyes. Adolescents with BPD do not always recover, even if they frequently shed certain BPD-related features with time. Any personality disorder with high levels of adolescent symptoms will negatively impact functioning over the course of the next ten to twenty years; these consequences are frequently more pronounced or persistent than those associated with disorders associated with Axis I [20]. A spectrum connection between borderline personality disorder and schizophrenia was implausible. Except for the periodic lapses in reality testing, borderline patients were intensely emotional and interpersonally needy [21]. Compared to women with borderline personality disorder (BPD) or bulimia nervosa/bulimia spectrum disorder (BN/BSD-BPD), women with BN/BSD-BPD showed significantly lower levels of serotonin and monoamine oxidase activity (HC). When compared to women with BN/BSD and HC, women with BN/BSD-BPD also showed higher levels of brain-derived neurotrophic factor, alterations in the methylation of the glucocorticoid receptor gene promoter (NR3C1), and dopamine receptor gene promoter methylation [22]. Both complex post-traumatic stress disorder (cPTSD) and perinatal BPD are linked to severe impairments in interpersonal functioning and an increased likelihood of psychopathology being passed down through generations [23].

Diagnosis of borderline personality disorder

BPD, a mental health disease that begins in early adulthood, is characterized by chronic instability in relationships, one's self-image, emotions, and impulsive conduct. At least five of the given nine signs must be present for a person to be diagnosed with the condition: ongoing empty feelings, intense or inappropriate anger, fear of abandonment, unstable interpersonal relationships, identity confusion, impulsivity, recurrent suicidal thoughts, emotional instability, and occasionally psychotic-like thinking or dissociation [24]. BPD covers a wide range of emotional and social difficulties. The dread of abandonment, unstable interpersonal connections that oscillate between idealization and devaluation, a shaky self-image, impulsive behaviors, and frequent suicidal thoughts are among its main symptoms. Rapid mood swings, emptiness, unrestrained wrath, and occasionally skewed perceptions or dissociation are additional symptoms that BPD sufferers may encounter [25]. These symptoms highlight the difficulties BPD sufferers have. Symptoms of BPD are shown in Table 1.

Symptoms of BPD

**Table 1:** Symptoms of BPD BPD: borderline personality disorder

Symptom number	Description of symptom
i	Fear of abandonment
ii	Unstable intimate relationships
iii	Identification disorder
iv	Impulsivity
v	Repeated suicide attempts
vi	Emotional instability
vii	Feelings of desolation that persist
viii	Severe, unreasonable rage
ix	Extreme dissociation or quasi-psychotic thoughts

Treatment of borderline personality disorder

The most commonly given drugs for BPD are fluoxetine, selective serotonin reuptake inhibitors (SSRIs), and citalopram, despite a shortage of research to support their usage [26]. It was shown that the most common correlation between giving antidepressants to individuals with BPD is comorbidity for affective disorders [26]. Early childhood impacts on the development of BPD's psychopathology include parenthood-related issues such as dysfunctional parenting, parenting philosophies, and parenting psychopathology [27]. Staged therapy designs, additional treatments, and technology-based interventions could be beneficial in cases where developing cost-effective interventions is necessary when people are newly diagnosed or are awaiting full-package therapy (or both) or when a particular deficit needs to be addressed in the context of ongoing treatment [28]. Furthermore, considering the growing body of research indicating that BPD is a diagnosable disorder in teenagers, treatments aimed at younger demographics constitute an important and required step. Table 2 displays the treatments available for BPD.

**Table 2:** Treatments of BPD DBT: dialectical behavior therapy; MBT: mentalization-based therapy; TFP: transference-focused psychotherapy; ST: Schema therapy

Type of treatment	Description	Purpose/Outcome
Psychotherapy		
Dialectical behavior therapy (DBT)	A type of cognitive-behavioral therapy explicitly developed for BPD. It incorporates interdependence, emotion control, awareness, and distress tolerance.	Addresses self-harm behaviours, improves emotional regulation and enhances interpersonal relationships.
Mentalization-based therapy (MBT)	It focuses on recognizing and understanding the feelings and thoughts in oneself and others.	It helps patients understand their own emotions and the emotions of others, improving interpersonal relationships.
Transference-focused psychotherapy (TFP)	Focuses on understanding and resolving emotions and interpersonal issues through the patient-therapist connection.	It helps individuals understand their emotions and change problematic patterns of interaction.
Schema therapy (ST)	Combines elements of cognitive, behavioural, and psychodynamic therapies. Focuses on changing negative life patterns.	It aims to identify and change maladaptive life patterns.
Medications		
Antidepressants	Includes selective serotonin reuptake inhibitors (SSRIs) and others.	It can help with mood swings, irritability, and feelings of emptiness.
Antipsychotics	They were often used in low doses.	It can reduce symptoms of anger, impulsivity, and brief psychotic episodes.
Mood stabilizers	Such as lithium or certain anticonvulsant medications.	It can help stabilize mood swings and reduce impulsivity.
Other treatments		
Hospitalization	They were often used in severe symptoms or if there's a risk of self-harm.	Provides a safe environment for stabilization and intensive treatment.
Group therapy	It provides a place to share experiences and coping techniques.	Encourages understanding and support among peers with similar challenges.

Psychotherapy, such as dialectical behavior therapy (DBT) for emotional regulation and relationship improvement and mentalization-based therapy (MBT) to understand one's own feelings as well as those of others, is the mainstay of treatment for BPD [29]. While Schema therapy targets dysfunctional life patterns, transference-focused psychotherapy (TFP) uses therapist-patient interaction to gain emotional insight [8]. Antipsychotic drugs control rage and impulsivity; mood stabilizers target mood swings; and antidepressants handle mood and irritability [8]. Hospitalization guarantees safety and intensive care in severe conditions. In group therapy, participants foster peer support and develop common coping mechanisms. Before beginning any treatment, it is essential to seek professional guidance. Borderline personality disorder (BPD) often manifests throughout adolescence; early identification and treatment are crucial [8,29]. While many BPD sufferers notice considerable improvements with time, it's essential to remember that a sizable percentage may continue to struggle with lingering symptoms as they age [30]. For prognosis, treatment planning, and patient counseling, being aware of these little differences is crucial from a professional standpoint. The analysis of the studies included is shown in Table 3.

**Table 3:** Analysis of the studies included EMDR: eye movement desensitization and reprocessing; DBT: dialectical behaviour therapy; PTSD: post-traumatic stress disorder

Author(s)	Year	Main characteristics
Mezei et al. [1]	2020	1. Examines BPD through a developmental psychopathology lens.
Zimmerman et al. [2]	2013	2. Investigates the connection and distinctions between BPD and bipolar disorder.
Paris et al. [3]	2005	3. General overview, diagnosis, and management of BPD.
Paris et al. [4]	2019	4. Probes into suicidality and its links with BPD.
Videler et al. [5]	2019	5. Discusses BPD's manifestations across various life stages.
Gartlehner et al. [6]	2021	6. Comprehensive analysis of pharmaceutical interventions for BPD.
Parker et al. [7]	2019	7. Overview of pharmacological strategies for treating BPD.
Stoffers et al. [8]	2012	8. Evaluates the effectiveness of psychological therapies for BPD.
Helle et al. [9]	2019	9. Examines how antisocial and borderline personality disorders can coexist with alcohol use disorder.
Reichl et al. [10]	2021	10. Focuses on self-harm behaviours within the BPD context.
Kienast et al. [11]	2014	11. Delves into the epidemiology and treatment of BPD coexisting with addiction.
Cremers et al. [12]	2021	12. Uses brain network measures to classify BPD during emotion regulation tasks.
Cavelti et al. [13]	2021	13. Looks at the emergence of psychotic symptoms in BPD from a developmental perspective.
Guendelman et al. [14]	2014	14. Investigates the neurobiological underpinnings of BPD.
Oud et al. [15]	2018	15. Systematic review of specialized psychotherapies for adult BPD patients.
Snoek et al. [16]	2020	16. A study comparing the costs and benefits of integrated EMDR-DBT with EMDR for PTSD patients who exhibit characteristics of BPD.
Biskin et al. [17]	2012	17. Discusses the diagnostic criteria and methods for BPD.
Masland et al. [18]	2020	18. Reconsiders the significance of boredom as a potential diagnostic criterion for BPD.
Tan et al. [19]	2018	19. Uses a qualitative approach to understand patients’ perceptions of schema therapy for BPD.
Larrivée et al. [20]	2013	20. Discusses challenges and peculiarities of diagnosing BPD in adolescents.
Gunderson et al. [21]	2009	21. Chronicles the development and changes in the diagnosis of BPD over time.
McDonald et al. [22]	2019	22. A thorough analysis of the genetics, epigenetics and comorbidity of BPD and bulimia nervosa.
May et al. [23]	2023	23. Reviews interventions for borderline personality disorder and complex trauma in the perinatal period.
Lekgabe et al. [24]	2021	24. Examines traits of BPD in adolescents suffering from anorexia nervosa.
Symptoms - borderline personality disorder [25]		25. An online resource detailing the symptoms of BPD.
Pascual et al. [26]	2023	26. Discusses pharmacological approaches to BPD and its frequently co-occurring disorders.
Kaur et al. [27]	2023	27. Investigates the influence of parenting in the onset and development of BPD.
Temes et al. [28]	2019	28. Offers insights into recent advances in psychosocial interventions for BPD.
Mayo Clinic [29]	2022	29. A resource from the Mayo Clinic detailing diagnosis and treatment modalities for BPD.
Biskin et al. [30]	2015	30. Explores the progression and lifetime trajectory of BPD.

## Conclusions

Understanding and managing BPD remains one of the most intricate and challenging mental health issues. Due to its unpredictable moods, behaviors, and interpersonal ties, diagnosis and treatment must take a multidimensional approach. The interconnectedness of BPD with other diseases, like bipolar disorder and psychotic symptoms, emphasizes its complexity and necessitates a comprehensive approach to therapy. Although its origins are unclear, the focus on symptoms provides professionals with a clear path for diagnosis. Recent developments in psychotherapy approaches, particularly Schema therapy and dialectical behavior therapy (DBT), have given individuals suffering new hope by uncovering viable routes to recovery. Although experts frequently recommend some pharmacological therapies, ongoing controversy about their effectiveness emphasizes the need for further study in this field.

The high rate of self-harming behaviors and suicidal thoughts among BPD patients is an important cause for concern, underscoring the importance of early detection, particularly in younger groups. Early intervention can significantly change the trajectory of the condition, which is why the urgency is increased by the fact that BPD frequently manifests during adolescence. In addition, the historical context, from Stern's 1938 coining of the word “borderline” to the complexity of today's diagnosis and treatment of an illness, has drawn growing attention over the years. Significant obstacles still exist. A more complex knowledge of BPD is crucial as we go. To guarantee people afflicted by BPD receive the comprehensive support they require, ongoing research, interdisciplinary collaboration, and patient-centered care are essential.
